# Palliative care for adolescents and young adults with advanced illness: A scoping review

**DOI:** 10.1177/02692163221136160

**Published:** 2022-11-09

**Authors:** Mohamed Abdelaal, Jonathan Avery, Ronald Chow, Nasreen Saleem, Rouhi Fazelzad, Pamela Mosher, Breffni Hannon, Camilla Zimmermann, Ahmed al-Awamer

**Affiliations:** 1 Division of Palliative Care, Department of Medicine, University of Ottawa, Ottawa, ON, Canada; 2 Ottawa Hospital Research Institute, Ottawa, ON, Canada; 3 Department of Supportive Care, Princess Margaret Cancer Centre, University Health Network, Toronto, ON, Canada; 4 Division of Palliative Medicine, Department of Medicine, University of Toronto, Toronto, ON, Canada; 5 School of Nursing, University of British Columbia, Vancouver, BC, Canada; 6 Temerty Faculty of Medicine, University of Toronto, Toronto, ON, Canada; 7 UHN Library and Information Services, Princess Margaret Cancer Centre, University Health Network, Toronto, ON, Canada; 8 Division of Child and Adolescent Psychiatry, Hospital for Sick Children, Toronto, ON, Canada; 9 Department of Psychiatry, University of Toronto, Toronto, ON, Canada; 10 Division of Palliative Care, Department of Family and Community Medicine, University of Toronto, Toronto, ON, Canada

**Keywords:** Adolescents, young adults, symptom management, psychosocial, palliative care, end-of-life

## Abstract

**Background:**

Age-related complex medical conditions have been commonly reported among adolescents and young adults with advanced life-limiting illness. There is increasing interest in exploring their palliative care needs and end-of-life experiences.

**Aim:**

This scoping review aimed to explore the available literature about providing palliative and end-of-life care to adolescents and young adults with advanced life-limiting illnesses.

**Design:**

Scoping review. This review was registered on Open Science Framework (https://doi.org/10.17605/OSF.IO/SPTD7).

**Data sources:**

Electronic databases (MEDLINEALL, Embase, Emcare, Cochrane Central Register of Controlled Trial CENTRAL, Scopus, PsycINFO, Cochrane Database of Systematic Reviews), Google Scholar and reference lists were searched up to October 2021. We included studies reporting on adolescents and/or young adults with advanced life-limiting illnesses. There were no limitations concerning location, type of illness or study design.

**Results:**

We identified 51 studies published between 2002 and 2021. Most studies were published in the United States (*n* = 34, 67%), and nine studies (18%) reported exclusively on patients with non-malignant illnesses. Two thirds of the identified studies were case reports and retrospective chart reviews (*n* = 33). Three main topics were identified: Physical symptom burden (*n* = 26, 51%), Psychological and social needs (*n* = 33, 65%), and end-of-life care (*n* = 30, 59%). Twenty-six studies (51%) were focused only on one topic, and the age range used to identify adolescents and young adults varied based on the study location.

**Conclusion:**

The findings of this review shed light on the different palliative care experiences and knowledge gaps related to adolescents and young adults as an underserved and vulnerable patient population. Further research needs to be dedicated toward palliative care programs tailored for adolescents and young adults.

**What is already known about this topic?**

- Palliative care integration is associated with better quality of life and improved end-of-life care
- Clinical guidelines recommend the early integration of palliative care to standard medical care.
- The availability of palliative care support designed for adolescents and young adults is still limited.

**What this review adds?**

- Complex symptom management burden, and high psycho-social needs are characteristic for adolescents and young adults with advanced life-limiting illness.
- Many adolescents and young adults with advanced disease have late or no access to palliative care consults before end of life
- High-intensity medical care at the end-of-life is more common among adolescents and young adults with hematological malignancies, and results in poor quality of end-of-life care.

**Implications for practice, theory or policy**

- There is a lack of global collaboration to support the palliative care needs of adolescents and young adults with advanced disease
- Research on how to improve adolescents and young adults’ access to palliative care services is urgently needed.

## Introduction

Patients with life-limiting illnesses face many challenges due to the physical burden of their illness, treatment side effects, and the social and psychological burden of living with their disease.^1^ Palliative care, as defined by the World Health Organization (WHO), is an approach that aims to improve the quality of life of patients and their caregivers.^2^ It aims to support patients with life limiting illnesses,^3,4^ by providing physical, functional, spiritual, and psychosocial support.^5,6^ Randomized controlled trials and meta-analyses in adults have demonstrated that early palliative care integration leads to better quality of life, lower acute health care utilization, and improved advance care planning.^7,8^

Adolescents and young adults have unique medical, psychological, social, and supportive care needs that differ from those of children and older adults.^9,10^ Within palliative care, there is no consistent international age range defined for adolescent and young adults. In North America, the age range is defined as being from 15 to 39 years old.^11^ There are different causes of disease-related mortality among this age group, but cancer remains the most common,^12–14^ with increasing annual incidence since 1992.^14,15^ Other life-limiting diseases that occur in the adolescent and young adult age group include cardiac diseases, liver cirrhosis, cerebrovascular diseases, and human immunodeficiency virus (HIV).^14^

Despite clinical guidelines and recommendations for early palliative care referrals,^16^ the integration of age-designated palliative care programs, provided by medical teams with special training in palliative care for adolescents and young adults, remains limited.^17^ Research on the palliative care needs of adolescent and young adults is also still developing, with scarce available data.

Scoping reviews, as a structured evidence synthesis process, serve to identify the scope of the literature on a certain topic, rather than to produce a synthesized answer to a specific question.^18^ Our scoping review aimed to explore the current data relevant to the palliative and end-of-life care of adolescents and young adults diagnosed with advanced and life-limiting illnesses. The primary objective was to delineate the existing knowledge regarding palliative and end-of-life care for adolescents and young adults. The secondary objectives were to identify the different age ranges used to define adolescents and young adults as described in the relevant studies, and to explore the level and timing of palliative care integration for adolescents and young adults through examining referral patterns to palliative care.

Our aim was to describe the extent and nature of research on this topic, in order to better understand the specific palliative care needs of adolescents and young adults, improve palliative care service provided to this population, highlight any gaps in the literature, and identify opportunities for future research.

## Methods

### Summary

The protocol for this review was previously registered on Open Science Framework (https://doi.org/10.17605/OSF.IO/SPTD7).

This review used the five-stage method previously outlined by Arksey and O’Malley,^19^ with recommendations proposed by Levac et al.^20^ First, the research questions were defined and stated as objectives. Second, relevant studies were identified through a comprehensive search of electronic databases. Third, appropriate studies were selected from the search results. Fourth, the data were charted to describe the basic parameters of selected studies. Fifth, data were synthesized through summarizing the results.

### Research questions

The primary research question framing this scoping review was: what are the palliative and end-of-life care needs and experiences of adolescents and young adults with advanced life-limiting illnesses? The secondary research questions were: What are the age ranges used to identify adolescents and young adults? What are the referral practices to palliative care related to adolescents and young adults, with respect to referral frequency and timing?

### Search strategy

In collaboration with the University Health Network information specialist at Princess Margaret Cancer Centre, an extensive literature search was conducted from the databases’ inception to 21st October 2021, in Medline ALL (Medline and Medline Epub Ahead of print and In-Process & Other Non-Indexed Citations), Embase, Emcare, Cochrane Central Register of Controlled Trials, Cochrane Database of Systematic Reviews, and PsycInfo all from the OvidSP platform, and Scopus from Elsevier.

Search terms included “pain management,” “symptom management,” “symptom prevalence,” “psychosocial needs,” “hospice,” “end-of-life,” and “palliative care.” Where available, both controlled vocabulary terms and text words were used. Where applicable, the search was restricted to the English language, human studies, adolescents and “young adults and adults” based on each database indexed age group. The indexed age group was from 13 to 44 years for Medline, and 13 to 64 years for Embase, Emcare, and PsycInfo. (Supplemental Appendix- Medline ALL search).

Google Scholar and reference lists from included scoping and systematic review studies were searched by the research team to identify additional relevant studies.

### Study eligibility criteria

The inclusion and exclusion criteria for this review (Table 1) followed the Joanna Briggs Institute Reviewers’ Manual for scoping review methodology.^21^ We included studies that reported on adolescents and young adults who were receiving palliative or end-of-life care for any advanced life-limiting illness,^22^ including cancer and end stage organ failure. As the definition of the age range of adolescents and young adults varies among countries, only studies addressing the terms “Adolescent” and/or “Young Adult,” regardless of the age range, were included. There were no limitations related to study location, design, or sample size. Studies were excluded if adolescents and young adults were not specifically identified within the study participants or if the focus was on the needs of caregivers, families, or healthcare professionals rather than on patients themselves.

**Table 1.** Study eligibility criteria.

Eligibility	Inclusion criteria	Exclusion criteria
Population	Adolescents and young adults Receiving palliative or end-of-life care Any advanced stage life-limiting illness, malignant and non-malignant: • Defined as any condition that is known to be incurable, and death can be reasonably expected within less than two years.^22^ Any racial, religious, cultural, political, economic or gender-based populations Any setting (acute care, community dwelling etc.) Any healthcare system (private or public) Studies with any number of subjects	Studies not including the terms “Adolescent” or “Young Adult” in their population. If the study participants include adolescents and/or young adults as part of a larger mixed-age sample, but without clear identification of the relevant findings from the adolescents and young adults, then group these studies separately. Studies focusing on the needs/challenges of caregivers, families, healthcare providers and/or their educational needs
Intervention	Palliative care intervention was defined as any of the following: • symptom-related intervention (symptom prevalence/intensity/management) • social, spiritual, psychological intervention, including non-pharmacological therapy • advanced care planning and discussions around this topic End-of-life care was defined as any medical service involving patients during the last days of life who are approaching anticipated death.	
Study design	Quantitative and/or qualitative studies Observational (e.g. surveys, focus groups, interviews, ethnographies/field studies, questionnaires) or experimental (e.g. randomized control trials) With and without control or comparison groups Studies with control groups, any form of control was considered Studies from any country of origin	Studies in languages other than English Opinion letters, and studies without patient population Studies in protocol form only Scoping and systematic reviews were excluded, but their reference lists were searched manually

Articles were independently reviewed in-duplicate by two of four reviewers (MA, JA, RC, NS), during Level 1 title & abstract and Level 2 full text screening. A calibration exercise of 20 articles was conducted before each screening level to ensure sufficient agreement (at least 80%) between the investigators. Disagreements were resolved by discussion and subsequent consensus of the two reviewers. If consensus was not reached, a third investigator (AA) was involved to determine whether the study met the criteria for inclusion.

### Data extraction and synthesis

Data extraction was performed in duplicate by two of four reviewers (MA, JA, RC, NS), with a third investigator (AA) involved to settle any disagreements. Data from each included study was documented using standardized forms, created and approved by all reviewers. The extracted data included: first author, year of publication, study type and design, study population, sample size and the age range used, type of the advanced illness, palliative care referral patterns (if available), and key findings. Data synthesis, including identifying key findings domains, and highlighting the gaps in knowledge, was completed by the reviewers. The final results are presented as tables and detailed descriptions.

## Results

**Figure 1.**
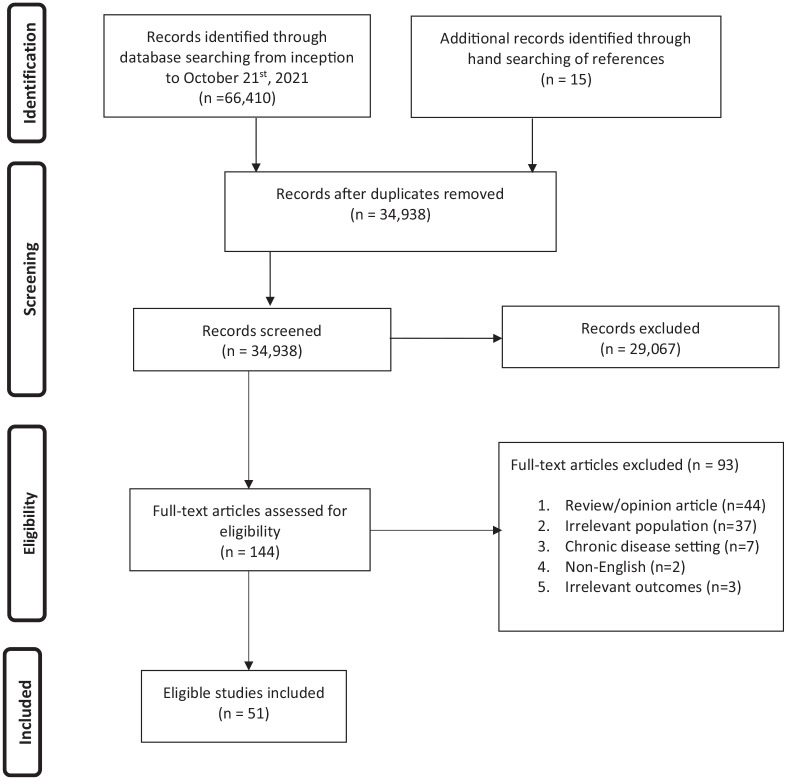
PRISMA flow diagram.

A total of 66,425 records were identified through searching the electronic databases, grey literature, and manually searching the references including the relevant scoping and systematic reviews (Figure 1: PRISMA diagram). No relevant abstracts were identified in the grey literature. After removal of duplicates, 34,938 abstracts were screened, and 144 full articles were assessed for eligibility. Ultimately, 51 studies, including 57,116 patients from the adolescent and young adult age group, were included.

The general characteristics of these studies are summarized in Table 2. Most of the studies were published in the US (*n* = 34, 67%), and we were unable to identify any relevant studies published before 2002. The majority were quantitative studies (*n* = 24, 47%), or case reports (*n* = 15, 29%). Most reported exclusively on adolescents and young adults (*n* = 44, 86%) and included exclusively patients with cancer (*n* = 42, 82%). However, four studies included only patients with non-malignant advanced illnesses (HIV/AIDS in three,^23–25^ and cystic fibrosis in one^26^). Five studies included patients with both malignant and non-malignant advanced illnesses.^27–31^

**Table 2.** General characteristics.

Author	Location	Study type, design	Population	AYA sample size	Age range (in years)	Type of advanced illness	Age-specific care
Abu-Saad Huijer et al.^55^	Lebanon	Quantitative, cross-sectional	AYA and children	46	Adolescents: 13–18	Cancer	Pediatrics/Children
Albrecht et al.^67^	US	Qualitative, longitudinal	AYA and adults	7	YAs: 18–39	Cancer (Leukemia)	Adults
Bell et al.^32^	US	Quantitative, chart review	AYA only	103	Adolescents: 10-21	Cancer	N/A
Cohen-Gogo et al.^42^	France	Quantitative, chart review	AYA only	45	AYA: above 13 years	Cancer	Pediatrics and Adolescents
Erickson et al.^56^	US	Quantitative-qualitative, longitudinal	AYA only	85	Adolescents and “emerging adults”: 15-29	Cancer	Pediatrics/Children’s Sites in three out of five recruitment sites
Flavelle^33^	Canada	Case report	AYA only	1	Adolescents: 10-18	Cancer (Sarcoma)	Pediatrics/Children
Fletcher et al.^51^	Australia	Quantitative, chart review	AYA and adults	27	AYA: 15-25	Cancer	N/A
Paice et al.^57^	US	Case report	AYA only	1	N/A	Cancer (Neuroblastoma)	Adults
Hughes et al.^50^	Australia	Quantitative, chart review	AYA and adults	33	AYA: 15–25	Cancer	Adolescents, Young Adults, and Adults
Hugo and van der Merwe^26^	South Africa	Qualitative, interviews	AYA only	6	Middle and late adolescence: 15–22	Cystic fibrosis	N/A
Humphrey and Lynn Dell^53^	US	Case report	AYA only	1	N/A	Cancer (Leukemia)	Pediatrics/Children
Johnson et al.^58^	US	Case report	AYA only	1	N/A	Cancer (Leukemia)	Pediatrics/Children
Johnston et al.^39^	US	Quantitative, chart review	AYA only	12,883	AYA: 15-39	Cancer	Pediatrics/Children’s sites + Adults’ sites
Keim-Malpass et al.^36^	US	Quantitative, chart review	AYA only	61	YAs: 18-39	Cancer	Adults
Klepping^35^	UK	Case report	AYA only	1	YAs: 16-35	Cancer (Nasopharyngeal)	Young Adults
Knox et al.^38^	Canada	Qualitative, interviews	AYA only	10	AYA: 15-39	Cancer	Adults
Ladas et al.^59^	US	Quantitative, chart review	AYA only	4	N/A	Cancer	Pediatrics/Children
Lau et al.^49^	US	Quantitative, RCT	AYA only	92	AYA: 12-25	Cancer	Pediatrics/Children
Lyon et al.^25^	US	Quantitative, questionnaire	AYA only	48	Adolescents: 14-21	HIV/AIDS	Pediatrics/Children
Mack et al.^40^	US	Quantitative, chart review	AYA only	111	AYA: 15-39	Cancer	N/A
Mark et al.^37^	US	Quantitative, chart review	AYA only	71	YAs: 18-39	Cancer	Pediatrics/Children
Nayak and Salins^60^	India	Case report	AYA only	1	N/A	Cancer (Head and neck)	N/A
Needle et al.^27^	US	Qualitative, questionnaire	AYA only	10	AYA: 14-27. Adolescents: 14-17. YAs: 18-27	Cancer and Benign hematological disorders	Pediatrics/Children
Peck et al.^61^	US	Case report	AYA only	1	N/A	Cancer (Sarcoma)	Pediatrics/Children
Penson et al.^68^	US	Case report	AYA only	1	N/A	Cancer (Leukemia)	Adults
Phenwan^62^	Thailand	Case report	AYA only	1	N/A	Cancer (Lymphoma)	Adults’ site, before transfer to Pediatrics/Children’s site
Poort et al.^54^	US	Quantitative, chart review	AYA only	138	YAs: 18-34	Cancer	N/A
Wiener et al.^30^	US	Quantitative-qualitative, interviews	AYA only	52	AYA: 15-34	Cancer and HIV	Pediatrics/Children’s site in one out of two recruitment sites
Roeland et al.^41^	US	Quantitative, chart review	AYA only	252	AYA: 15-39	Cancer	N/A
Satija et al.^63^	India	Case report	AYA only	1	N/A	Cancer (Head and neck)	Adults
Selwyn and Forstein^23^	US	Case report	AYA only	1	N/A	HIV/AIDS	N/A
Sison et al.^69^	US	Case report	AYA only	1	N/A	Cancer (Brain)	N/A
Snaman et al.^29^	US	Quantitative, chart review	AYA only	69	AYA: 15-39	Cancer and Aplastic Anemia	Pediatrics/Children
Rajeshuni et al.^43^	US	Quantitative, chart review	AYA only	30,573	AYA: 15-39	Cancer	N/A
Tenniglo et al.^64^	Netherlands	Qualitative, questionnaire	AYA and adults	11	N/A	Cancer	Pediatrics/Children
Veneroni et al.^70^	Italy	Case report	AYA only	1	N/A	Cancer (Sarcoma)	N/A
Wiener et al.^31^	US	Quantitative-qualitative, interviews	AYA only	20	N/A	Cancer and HIV	Pediatrics/Children
Poort et al.^52^	Netherlands	Quantitative, questionnaire	AYA and adults	83	AYA: 18-35	Cancer	Adolescents and Young Adults
Lyon et al.^24^	US	Quantitative, interviews	AYA only	38	Adolescents: 14-21	HIV/AIDS	Pediatrics/Children
Snaman et al.^28^	US	Quantitative, chart review	AYA only	69	AYA: 15-39	Cancer and Aplastic Anemia	Pediatrics/Children
Levy et al.^65^	US	Case report	AYA only	1	N/A	Cancer (Neuroblastoma)	Pediatrics/Children
Abdelaal et al.^44^	Canada	Quantitative, chart review	AYA only	69	AYA: 15-39	Cancer	Adults
Kassam et al.^45^	Canada	Quantitative, chart review	AYA only	7122	AYA: 15-39	Cancer	N/A
Lockwood et al.^46^	US	Quantitative, chart review	AYA only	4674	AYA: 15-39	Cancer	Adults
Avery et al.^71^	Canada	Qualitative, interviews	AYA only	12	YAs: 18-39	Cancer	Adults
Fladeboe et al.^48^	US	Quantitative-qualitative, interviews	AYA only	26	AYA: 12-24	Cancer	Pediatrics/Children
Currin-McCulloch et al.^72^	US	Qualitative, interviews	AYA only	13	YAs: 18-39	Cancer	N/A
Rosa et al.^66^	US	Case report	AYA only	1	N/A	Cancer	Young Adults
Trevino et al.^47^	US	Quantitative, interviews	AYA only	71	AYA: 15-39	Cancer	N/A
Taylor et al.^73^	US	Descriptive feasibility study	AYA only	85	N/A	Cancer	N/A
Labudde et al.^34^	US	Quantitative, chart review	AYA and Children	82	Adolescents: 13-18	Cancer	Pediatrics/Children

YAs: young adults; AYA: adolescents and young adults.

### Definition of age range

An age range was not defined in 17 studies (33%). In the remaining 34 studies, 19 different age ranges were defined: six for adolescents; four for young adults; and nine different age ranges for the studies that included adolescents and young adults.

The age used to define adolescents ranged from 10 to 22 years^26,32–34^;the age used to define young adults ranged from 16 to 39 years^35–37^; and the age used to define adolescents and young adults ranged from 13 to 39 years.^38–42^Most studies originating from Canada and the US used the age range of 15–39 years old^28,29,38–41,43–47^; however, other age ranges were also reported in US studies, including 12–24,^48^ 12–25,^49^ 14–27,^27^ and 15–34.^30^ Two studies published in Australia used a range of 15–25 years,^50,51^ a study from the Netherlands used 18–35,^52^ and a study from France defined only a lower limit of 13 years.^42^

### Race/Ethnicity

The correlation between race/ethnicity and palliative and end-of-life care was documented in seven studies.^24,34,39,41,43,47,49^ Patients of white race stated higher social support,^47^ were more likely to receive palliative care,^41^ and less likely to die in a hospital.^43^ African-American patients expressed higher interest to continue active treatments even if their physical ability was to decline.^24^ Patients of Hispanic ethnicity were more likely to die in a hospital,^43^ and had higher hospital bed utilization in the last year of life, compared to non-Hispanic ethnicity.^39^ The use of a resilience promoting intervention had a higher response in finding benefit among non-white patients, compared to white patients.^49^ In one study, there was no difference in the number of palliative care opportunities across racial and ethnic groups.^34^

### Palliative care referral practices

Twelve studies contained information on referral practices to palliative care among adolescents and young adults.^28,29,34,36,37,41,44–46,51,53,54^ The time between palliative care referral and death was reported in five studies,^34,36,37,44,51^ with the mean time ranging from 12.8 days^36^ to 94.4 days,^51^ and a median time of 53 days,^51^ 62 days,^34^ 5 months,^44^ and 318 days.^37^ One study reported the median time between cancer diagnosis and palliative care referral was 144 days among hematological malignancies, and 458 days among patients with neurological malignancies.^46^ Palliative care referral during the last month of life varied between 7% of the adolescents and young adults in one study,^37^ 17% in another,^54^ and 68% of the patients who received a stem cell transplant (compared to 77% in patients who did not receive a stem cell transplant).^29^ One study reported that 18% of adolescents and young adults had palliative care referral during the last day of life,^36^ whereas in another up to 43% of patients had no palliative care involvement.^54^ Patients with hematological malignancies were less likely to receive referral to palliative care or hospice.^36,41,45^

### Domains of palliative care needs and experiences

After analyzing the key findings of the 51 studies, three domains were identified related to the palliative care needs and experiences of adolescents and young adults: physical symptom burden (*n* = 26, 51%),^26,28,29,32–35,40,42,44,46,50,52,53,55–66^ psychological and social needs (*n* = 33, 65%),^26,28,29,31–33,35,38,40,42,44,46–50,53,55,57,58,60–64,66–73^ and end-of-life care (*n* = 30, 59%).^23–25,27–32,34–37,39–45,51,53,54,58,60,62,65,66,68^ The three domains were all presented in 13 articles (25%). Twelve articles (24%) discussed two domains, and 26 articles (51%) focused on one domain only.

### Physical symptom burden

This domain was presented in 26 articles, and can be further divided into symptom incidence, severity, and management (Table 3). Most articles (*n* = 15/26, 58%) discussed at least two of these topics, and all three topics were presented in three articles.^55,62,63^ In eight articles, only one topic was presented (Symptom incidence: *n* = 7,^26,33,34,40,46,52,56^ management: *n* = 1^32^).

**Table 3.** Physical symptom burden.

Author	Incidence	Severity	Management
Abu-Saad Huijer et al.^55^	Tiredness reported in 63% of patients, pain and lack of appetite in 54%	MSAS Pain mean score: 2.54	Most common treated symptoms: vomiting (82%), cough (70%), pain (68%) and nausea (67%) with success rate of 67%, 57%, 59% and 50% respectively
Bell et al.^32^			87% of the patients used pain medications, and 46% used anti-emetics for nausea and vomiting
Cohen-Gogo et al.^42^	Median number of physical symptoms at EOL was 4. Pain and dyspnea were the most common report symptoms		40% of the patients had refractory symptoms including pain and dyspnea. Opioids were used in 84% of the patients for pain, morphine being the most common
Erickson et al.^56^	Most common reported symptoms: tiredness (42%), nausea (39%), pain (37%), drowsiness (33%)		
Flavelle^33^	Pain and tiredness were the most limiting symptoms, and increasing toward the last 4 weeks of life		
Levy et al.^65^	Severe pain was reported at EOL		Pain was not well managed with self-administering medications. Methadone was not suggested due to patient’s non-compliance with opioids. Fentanyl patch was used with little effect. Patient was reluctant to take pain medications
Paice et al.^57^	Severe pain was reported, and might have been aggravated by anxiety and existential distress		Opioids, up to 800 mg MEDD, were used for pain management. Opioid addiction concerns were raised by the patient, and methadone was declined due to stigma
Hughes et al.^50^	Most common reported symptoms: pain (91%), wellbeing (76%), tiredness (75%) and lack of appetite (67%)	ESAS pain median score: 6, with 33% reporting moderate and 36% reported severe pain.	
Hugo and van der Merwe^26^	Tiredness, insomnia, lack of appetite, and dyspnea were reported		
Humphrey and Lynn Dell^53^	Pain secondary to mucositis was reported		Opioids were used to manage the pain. Concerns about chemical coping and substance misuse were raised by the treating physicians
Johnson et al.^58^	Severe pain was reported		Opioids, up to 4000 mg MEDD, were used to manage the pain with little effect. Palliative sedation was suggested
Klepping^35^	Severe total pain was reported		A holistic approach to manage total pain was used including: opioids, adjuvants, TENS, massage, relaxation, and distraction
Ladas et al.^59^	Patients reported bleeding from tumor sites and uncontrollable epistaxis		Patients reported bleeding control improved with the addition of Yunnan Baiyao (Chinese herbal medicine) to conventional hemostatic interventions.
Mack et al.^40^	Most common symptoms during the last month of life: pain (94%), fatigue (79%), nausea (68%)		
Snaman et al.^28^	Most common reported symptoms during the last month of life: pain (99%), fatigue (85%), edema (78%).		97% of patients received opioids during the last month of life. 86% of patients had at least one documented refractory symptom
Nayak and Salins^60^	Uncontrolled pain was reported at the initial palliative care visit		Pain was optimally controlled with oral analgesics administered through Ryle’s tube
Peck et al.^61^	Severe pain was reported, and might have been aggravated by anxiety		Opioids, up to 113 mg MEDD, were used to manage pain, and aberrant opioid behavior was reported. A multimodal treatment approach was developed to control pain and anxiety using opioids, diaphragmatic breathing, cognitive restructuring, positive self-statements and guided imagery. This was associated with good effect and reduction in opioid use.
Phenwan^62^	Total pain was reported with all its aspects: physical, psychological, social and spiritual	Severe pain rated 9/10	A multimodal treatment approach was used including opioids, benzodiazepines and antipsychotics for anxiety, and social engagement in different activities
Poort et al.^52^	Fatigue was reported in 48% of the patients, and was associated with lower quality of life		
Satija et al.^63^	Severe pain was reported	Pain score was initially 9/10, reduced to 6/10 after nerve block but remained difficult to control	Pain was not relieved with weak opioids. Morphine, up to 120 mg, with adjuvants were used. Radiofrequency ablation was done with partial relief. Scrambler therapy and Flupirtine were also tried.
Snaman et al.^29^	Most common reported symptoms during the last month of life: pain (97%), fatigue (91%), edema (82%)		97% of patients received opioids for symptom management during the last month of life, and 79% had at least one documented refractory symptom
Tenniglo et al.^64^	Most common reported symptoms: fatigue and muscle weakness		
Abdelaal et al.^44^	Worst reported symptoms: tiredness, sleep, pain, drowsiness	84% had at least one moderate-to-severe symptom score. Most patients reported improved or stable symptoms after palliative care clinic visit	
Lockwood et al.^46^	Reasons for PC referral: pain (65%), constipation (41%), nausea (37%)		
Rosa et al.^66^	Reported severe pain		Opioid use was dependent on housing situation, and negatively affected by homelessness
Labudde et al.^34^	Adolescents experienced higher symptom burden compared to younger patients		

MSAS: Memorial Symptom Assessment Scale; TENS: transcutaneous electrical nerve stimulation.

Twenty five studies elaborated on the incidence of symptom among adolescents and young adults, whether during the illness trajectory or at end of life. The most prevalent symptoms were pain and tiredness.^29,40,50,55,56^ Pain was reported among 37%–91%^46,50,56^ of adolescents and young adults, and the incidence increased up to 99% of the patients during the last month of life.^28^ Similarly, tiredness was reported by 42%–75%^50,52,56^ of the patients, and increasing to 91% at end of life, with lower quality of life.^29,52^

Although the level of symptom severity was not regularly described in the reviewed studies, many case reports described severe pain in adolescents and young adults,^57,58,63^ rated at 9 out of 10 in different studies.^62,63^ Various techniques for symptom management were explained in some studies. However, optimal pain control was rare despite using high opioid doses,^57,61,63^ up to 4000 mg of morphine equivalent daily dose (MEDD).^58^ Concerns around opioid addiction, substance misuse and chemical coping were reported in two case reports.^53,57^

Total pain was described in three case reports^35,61,62^ where physical pain was aggravated by anxiety, or social distress. The use of benzodiazepines and antipsychotics, in addition to non-pharmacological interventions such as breathing techniques, were used to manage the pain in patients where opioids alone were not successful.^35,61,62^

### Psychological and social needs

Table 4 summarizes the 33 studies exploring the psychological and social needs for adolescents and young adults. Themes related to psychological symptom burden and adjustment disorders were discussed in 31 studies.^26,28,29,32,33,35,38,40,42,44,46–50,53,55,57,58,60–64,67–73^ During the last month of life, up to 87% of patients had at least one psychological symptom.^28^ Depression and anxiety were repeatedly reported by adolescents and young adults,^35,40,53,60,69^ in up to 43% and 48% of patients, respectively.^50^ The use of benzodiazepines was common, especially toward end of life.^28,58,62^ However, in the majority of cases, these psychological symptoms were either untreated,^40,53,55^ or refractory to treatment.^28,55^

**Table 4.** Psychological and social needs.

Author	Psychological	Social	Spiritual
Abu-Saad Huijer et al.^55^	Feeling irritable and worrying were among the most common reported symptoms. Feeling irritable was treated in 4% of patients with no success, while worrying was treated in 12% with 33% success rate		
Albrecht et al.^67^	Patients did their best to remain positive, but was difficult. Hearing the word “cancer” was difficult as it reflected isolation and lack of control. Patients wanted to hear positive things, but also expected more information from the healthcare teams	The role of family, friends, technology and social media were appreciated as tools of support	
Bell et al.^32^	More anti-anxiety medications were used among patients in late adolescence		
Cohen-Gogo et al.^42^	Sadness, anxiety, fear of being alone, fear of death, fear of pain and guilt were reported among 100% of patients		
Flavelle^33^	Fighting for independence, getting more responsible for health and wellbeing, and maintaining pride was reported by the patient, even if it meant not asking for help. Patient tried to find ways to escape from illness	Social relations were important, mainly family support. Patient avoided any romantic relationships due to his illness	Patient struggled to find meaning in life, but sought faith as source of strength
Paice et al.^57^	Anxiety and existential distress were reported, leading to increased pain scores. Distraction was used as a coping mechanism, and trust was essential for patient to express feelings		
Hughes et al.^50^	Depression and anxiety were reported in 48% and 43% of the patients respectively		
Hugo and van der Merwe^26^	The patients’ need to be understood and to understand was negatively affected as the illness affected socialization and learning. Loss and bereavement linked to illness was reported. Constructive internal dialog and positive thinking emerged as protective variables. Patients expressed hope for the future and found some meaning in the illness		Patients had questions about religion, but used faith as a major protective variable
Humphrey and Lynn Dell^53^	Major depressive disorder was reported, psychotherapy and antidepressants were recommended, but patient refused		
Johnson et al.^58^	Agitation and distress were reported, and treated with benzodiazepines and antipsychotics but with limited effect. Palliative sedation was offered		
Klepping^35^	Anxiety was reported, mainly around family’s feelings after patient’s death	Planning family visits helped in achieving better pain control for the patient	Patient got benefit from talking with a chaplain, despite being atheist
Knox et al.^38^	All patients described feelings of isolation, and not understood by others. Cancer was traumatic when it occurred in their young age. Most patients felt their necessary reliance on their parents, causing them to be developmentally delayed. Friends were key supports, while parents were typically considered default caregivers. Coping was done by engaging in activities and all patients identified individual psychotherapy as potentially important		
Lau et al.^49^	“Promoting Resilience in Stress Management” (PRISM) intervention improved resilience, quality of life and distress among patients, with small negative effect on distress among male patients		
Mack et al.^40^	Depression was reported by 41% of the patients, and only 43% of those with depression received treatment		
Snaman et al.^28^	87% of the patients had at least one psychosocial symptom during the last month of life with the median number of symptoms being three. 22% had at least one refractory psychosocial symptom, and 78% received benzodiazepines during the last month of life		
Nayak and Salins^60^	Most common reported thoughts: depression, anxiety, poor self-esteem, concerns about body image, and sense of isolation and separation		
Peck et al.^61^	Anxiety and panic attacks were reported. Anticipatory anxiety was also reported in relation to pain. Anxiety contributed to aberrant opioid behavior and opioids were used as a coping strategy		
Penson et al.^68^	Depression was reported. Other challenges were related to: loss of privacy, worry about younger sibling, fear of dying in pain. Humor was used as a coping strategy		
Phenwan^62^	Patient was diagnosed with adjustment disorder and suicidal tendencies, treated by benzodiazepines and antipsychotics. Moving the patient from adult to pediatric ward helped with adjustment	Social adjustment by contacting friends and teachers, and using social media was effective	
Satija et al.^63^	Psychological pain related to anxiety, deep stress, anger, guilt, helplessness and frustration was reported, and treated by psychological counseling	Worries about family and social status, and financial burden were described, and improved by open communication with hospital and home care teams	High spiritual distress was reported. Patient was blaming God for his sufferings
Sison et al.^69^	Severe anxiety was reported. Patient was reluctant to undergo treatment, and required Propofol sedation for agitation	Patient tried to hide her sufferings from parents by meeting with the healthcare team alone	
Snaman et al.^29^	Median number of psychological symptoms during the last month of life was three		
Tenniglo et al.^64^	Many patients searched for specialized psychological support, and felt psychosocial care should adapt to different needs based on age and phase of treatment	Patients’ ability to go to school was considered important for their social wellbeing	
Veneroni et al.^70^	Patient needed to talk about fear of dying, and was only able to trust the psychologist to express her feelings		
Wiener et al.^31^			Some patients wanted spiritual guidance, while others were uncomfortable. A suggestion was made to modify Five Wishes document and include an optional spiritual section
Abdelaal et al.^44^	Mean score for depression: 3.2, median: 2.5. Mean score for anxiety: 3.3, median: 3. After PC clinic visit, 39% had improved depression score, and 41% had improved anxiety score		
Lockwood et al.^46^	39% of patients were referred to PC for anxiety		
Avery et al.^71^	The word palliative was associated with death, dying and disease progression. Patients felt social stigma about being palliative. Patients felt PC clinic provided them with time explore their cancer experience, and respected the importance of including family support		
Fladeboe et al.^48^	Promoting Resilience in Stress Management–Advanced Cancer program to be feasible and highly acceptable. Resilience coaching followed by integrated ACP is feasible and acceptable for AYAs with AC. Participating did not cause distress or decrease hope		
Currin-McCulloch et al.^72^	Hope is an essential component in motivating Young Adults with cancer. “Contingent hope theoretical framework” describes the psychosocial behavior used by Young Adults, and consists of: navigating uncertainty, feeling broken, disorienting grief, finding bearings, and identity reconciliation		
Rosa et al.^66^		Being respected was a main demand by the patient	Patient was Christian, but his image of God was associated with abandonment and controlled anger. This was changed to a vision of a God that may care, after receiving appropriate medical, social and spiritual care
Trevino et al.^47^	Higher levels of total social support were reported among white patients, female patients, married patients, breast cancer patients, older ages. Higher levels of total social support were associated with better psychological and existential quality of life and less severe grief		
Taylor et al.^73^	Two potential methods for deriving heart rate variability that can be used as a psychosocial symptom biomarker in the palliative care setting		

SDM: substitute decision maker; DNR: do not resuscitate.

Emotional distress was another psychological challenge faced by the patients, and demonstrated in common terms such as “feeling isolated,” “loss of privacy,” and “fear of dying.”^38,42,60,67^ Patients in these studies used a variety of coping mechanisms including humor and engagement in different activities, such as with peer support groups.^38,57,68^

Additionally, the importance of social engagement with friends, and the role of family support constituted important coping tools for adolescents and young adults.^33,35,62,67^ The value of technology and social media was demonstrated as an effective way of keeping patients connected with their peers.^62,67^ On the other hand, adolescents and young adults in some studies preferred to hide their emotional distress from their parents,^69,70^ and avoided engaging in personal or romantic relationships during their illness.^33^

Spirituality and spiritual needs were explored in six studies.^26,31,33,35,63,66^ Some adolescents and young adults used their faith as a coping mechanism, despite their struggle to find meaning in life.^26,33^ However, other patients did not feel comfortable answering questions pertaining to their religious or spiritual beliefs.^31^

### End-of-life care

Components of advance care discussions and end-of-life care for adolescents and young adults were presented in 30 articles (Table 5). Code status decisions were discussed in 12 articles.^25,28,29,34,36,37,40,41,44,51,54,58^ While the majority of adolescents and young adults had “Do not Resuscitate” orders confirmed before death,^29,36^ the median time between this decision and time of death of some patients was as short as two days.^28^ A range of 1%–21% of adolescents and young adults had resuscitation attempted before death.^29,36,37,44,51^ Patients who received hematopoietic cell transplant, or those who were not followed by a palliative care team, were more likely to have resuscitation attempts at the end of life.^28,29^ In one study, 36%–44% of patients had either late or no discussions around code status, and 40% had no documented discussions around their goals of care.^54^

**Table 5.** End-of-life care.

Author	Code status	SDM	Goals of care	Chemo at EOL	Place of death	Other findings
Bell et al.^32^			12% had aggressive life sustaining measures		16% died at home, 33% in hospital, 23% in ICU	Time between EOL discussion and death ranged between 1-880 days
Cohen-Gogo et al.^42^				40% of patients received chemotherapy during the last month	13% died at home, 58% in hospital, 9% in a palliative care unit, 4% in ICU	33% were on artificial nutrition during the last week of life
Fletcher et al.^51^	7% of patients had DNR order, and 7% had resuscitation attempted at EOL	Parent (92.6%) or partner (7.4%)	52% of patients wanted active anti-cancer treatment, 7% wanted comfort measures.	41% received chemotherapy during the last month	74% wanted to be at home for end-of-life. However, 67% died at home, 22% in hospital, 7% in a palliative care unit	56% had at least one hospital admission during the last month of life, and 19% were on artificial nutrition during the last month of life
Humphrey and Lynn Dell^53^		Partner				
Snaman et al.^28^	87% of patients had DNR order (88% with PC intervention, and 84% without). 12% had resuscitation attempted (With PC: 8%, without PC: 21%)			49% received chemotherapy during the last month of life (With PC: 48%. Without PC: 53%)	54% died in an inpatient ward, and 46% in ICU (With PC: 62% in inpatient ward and 38% in ICU. Without PC: 32% in inpatient ward and 68% in ICU)	26% received dialysis (With PC: 22%, without PC: 37%). 71% received at least one medical procedure during the last month of life, with a median number of procedures: 3 (With PC: 1, without PC: 3). 52% of patients were on artificial nutrition
Johnson et al.^58^	Code status was “Do not Resuscitate”	Parent	Active medical management including antibiotics and parental nutrition, but forgo ICU admission			
Johnston et al.^39^						AYAs cancer patients spent a mean of 40 and 32 days in the hospital in their last year and 6 months of life respectively. Mean cost was $149,307 and $124,444 for the last year and 6 months of life respectively
Keim-Malpass et al.^36^	13% of patients had DNR order prior to hospital admission, 18% had resuscitation attempted and 88.5% confirmed DNR within a range of 1–60 days prior to death		33% had documented goals of care prior to admission, more among patients who did not have ICU admission		All patients in this study died in the hospital with 64% in an inpatient ward and 36% in ICU	Length of hospital stay was longer among patients with hematological malignancies, and those who had ICU admission at EOL. Patients who had palliative care visit more than one day before EOL had longer survival
Klepping^35^			Patient wanted comfort measures and no active medical management		Hospice was the place of death	
Lyon et al.^24^			Goals of care for the majority of patients were active medical interventions regardless of the quality of life			Anxiety and depression did not increase when having advance care planning discussions with patients, and quality of life was maintained
Mack et al.^40^	67% had DNR order, and 11% of these patients confirmed DNR before the last month of life		96% had documented goals of care discussions, 72% wanted active medical interventions early during the last month of life, reduced to 40% before death	11% received chemotherapy during the last two weeks of life	18% died at home, 27% in hospital, 14% in ICU, 2% in other locations and 40% were undocumented	78% received at least one intensive EOL measure during the last month of life, with 29% received ICU care, 30% had more than one emergency department visit, and 73% had at least one hospital admission
Mark et al.^37^	1% of patients had resuscitation attempted at EOL, while 79% had DNR order				32% of patients died at home, 52% in hospital, 3% in ICU, and 1% in a palliative care unit	Patients with hematological malignancies were less likely to enroll in hospice care
Nayak and Salins^60^					Home was the place of death	
Needle et al.^27^		Parents (90%)	Patients wanted to discontinue treatment if disease progression (70%) or poor quality of life (40%)			20% of the patients (*n* = 2) died, both were on mechanical ventilation, one had failed resuscitation, and the other died following parent’s decision to discontinue mechanical ventilation
Penson et al.^68^					Home was the place of death	Patient had ICU admission during the last month of life
Levy et al.^65^		Parents	Adequate pain and symptom management, and to die at home			Parents respected the patient’s autonomy and privacy, yet, did not offer any help with decision making or medication administration. This case raised concerns around the competency and level of maturity of this young patient to make own medical decision
Phenwan^62^					Home was the place of death	Patient had hospital admission during the last month of life
Poort et al.^54^	20% of the patients had early discussions about code status, 36% had late discussions, and 44% had no discussions		40% had early goals of care discussions, 20% had late discussions, and 40% had no discussions		17% died at home, 65% in hospital, 2% in a palliative care unit, and 17% in a nursing home	
Roeland et al.^41^	36% of the patients had DNR order			38% received cancer directed therapy during last month, 14% during last week of life	39% died in hospital	Patients living in poverty were less likely to receive early palliative care and more likely to receive inpatient palliative care
Wiener et al.^31^			40% found it stressful or very stressful to make medical decisions			95% found advance directive like Five Wishes to be helpful or very helpful to themselves, and 90% stated it would be helpful to others. No patients found it stressful
Selwyn and Forstein^23^		Aunt	Active medical management but to re-assess the condition if the patient deteriorates			
Snaman et al.^29^	82% of patients who received hematopoietic cell transplant (HCT) had DNR order at death			Among HCT patients: 29% received chemotherapy during the last month	Among HCT patients who died in the hospital, 29% died in an inpatient ward and 71% in ICU	82% of HCT patients received at least one medical procedure during the last month, with median number of procedures: 3. 82% of HCT patients were on artificial nutrition
Tenniglo et al.^64^			Patients appreciated autonomy in making decisions but relied on physicians’ advice in some topics such as choice of antibiotics for febrile neutropenia			
Wiener et al.^30^			83% thought it was important that “Five Wishes” or “My Thoughts, My Wishes, My Voice (MTMWMV)” be a legal document, 54% preferred MTMWMV, and 37% preferred Five Wishes			
Lyon et al.^25^	71% did not want life extending measures		71% feared painful death, and 8% feared addiction of pain medications		85% preferred to die at home, 39% would want hospice support	78% believed it was appropriate to initiate EOL decision making early in the course of their disease, 96% wanted honest answers from doctors, and 46% wanted to know about prognosis
Rajeshuni et al.^43^					33% died at home, 57% in hospital, 10% in other locations.	Younger patients (<30 years old), of minority race, of Hispanic ethnicity, who lived within 10 miles from a specialty center, and who had a diagnosis of leukemia or lymphoma were more likely to die in hospital
Abdelaal et al.^44^	4% had resuscitation attempted at EOL			6% received chemotherapy in the last 14 days of life	36% died in hospice/palliative care unit, 26% at home, 18% in inpatient ward, 12% in ICU	38% of the patients had emergency department visit, 48% had at least one hospital admission, 16% had ICU dmission in the last month of life
Kassam et al.^45^				9% received chemotherapy in the last 14 days of life	11% died in ICU	17% of patients had more than one emergency department visit. 22% had more than one hospitalization. 16% were admitted to the ICU. High intensity medical care was evident more among patients with hematological cancer
Rosa et al.^66^					Hospice was the place of death	
Labudde et al.^34^	61% of the patients had DNR order. Median time between DNR order and death: 6 days					40% of the patients were admitted to the ICU before death, and 6% had hospital admission for end of life

Moreover, some studies showed that the patients’ goals of care were mostly centered around active medical management,^23,24,40,51,58^ reaching up to 72% of the patients during the last month of life.^40^ However, many patients voiced their preference to discontinue treatment and pursue a comfort based medical approach if their prognosis was limited and before death.^27,40^ Generally, adolescents and young adults felt early discussions around goals of care and end of life were appropriate, helpful and did not affect their quality of life.^24,25,31^

Despite favoring to make treatment decisions, many adolescents and young adults found it stressful,^31^ and preferred to follow the medical teams’ advice.^64^ Additionally, opioid use was concerning to some patients, with fears of addiction.^25^ The choice of a substitute decision maker was another topic presented in six articles,^23,27,31,51,53,58^ and up to 93% of patients preferred to have their parents as their decision makers.^51^

More than one-third of adolescents and young adults received chemotherapy during the last month,^41,42,51^ and up to 14% during the last week of life.^41^ Similarly, more than 70% of patients received at least one medical intervention during the last month.^28,29,40^ In one study, 33% of the adolescents and young adults received artificial nutrition during the last week of life.^42^

Up to 85% of patients expressed a wish to die at home.^25,65^ However, inpatient hospital units were the most common place of death, ranging between 22% and 65% of patients.^37,40,51,54^ Patients with hematological malignancies, who received hematopoietic cell transplant, or who did not have palliative care involvement, were more likely to die in intensive care units (ICU).^28,29,32,36^

## Discussion

This scoping review identified 51 studies that addressed different aspects of palliative and end-of-life care for adolescents and young adults with advanced illness. Most of the studies (*n* = 45, 88%) were published within the last 10 years, and 67% originated from the US. More than two-thirds of the studies were retrospective chart reviews and case reports. These findings highlight the need for age-specific prospective studies that relate to adolescents and young adults, particularly in countries outside the US and for patients with non-malignant conditions.

### Age range

The age range used for adolescents and/or young adults was not identified in one third of studies. In the remaining studies, the age used to identify adolescents and young adults varied, with an interval from 10 to 39 years old among all the studies. However, this range was widely variable across the studies, and differed according to the country of origin, with North American studies tending to use the interval of 15 39 years,^28,29,38–41,43–47^ and studies originating in Australia tending to use the interval of 15–25 years.^50,51^ Indeed, definitions of the adolescent and young adult age group vary internationally, with the United States using a wider age range of 15 to 39 years, the UK uses 13–24 years, and Australia uses 15–25 years.^11^ Even within countries, the definition can vary; for example, the National Cancer Institute (NCI) in the United States used the age range of 15–29 years for clinical epidemiology purposes, but favored 15–39 years as a broader range for adolescent and young adult oncology.^11^ The differences in the age definition of adolescents and/or young adults across the literature creates discrepancies in identifying palliative and end-of-life experiences of this age group. These findings reflect the importance of defining a global age range for adolescents and young adults with advanced disease, in order to better understand their needs, and to more easily compare and improve the quality of palliative care programs for the adolescent and young adult population internationally.

### Complex symptom burden

Symptom control is one of palliative care’s main goals, which aims to improve quality of life.^74,75^ Adolescents and young adults with advanced illness face high symptom burden, with complex physical and psychological needs.^76–78^ This complexity is sometimes referred to as total pain, that can be refractory to high opioid doses.^57^ Adequate total pain management requires a multimodal approach including pharmacological and non-pharmacological interventions, as well as social and spiritual support.^35,62^

In addition, adolescents and young adults may express high levels of psychological and social distress, related to their stage of cognitive development, as well as a desire to preserve their privacy, along with worries about body image and financial burden.^79,80^ The majority of patients included in our review reported multiple psychosocial symptoms, and more than 20% were refractory to treatment.^28^ Tenniglo et al. highlighted the demand for specialized psychosocial care for adolescents and young adults, that needs to be compatible with the different needs of the adolescent and young adult age group.^64^

### Quality of end-of-life care

Some studies in our scoping review observed longer hospital admissions, less palliative care involvement and higher rates of ICU deaths among adolescents and young adults with hematological malignancies.^32,36,37,45^ Other factors related to the poor quality of end-of-life care included late palliative care referral, receiving a hematopoietic cell transplant, and patients’ financial status.^29,36,41^

People with cancer within the adolescent and young adult age group had high hospital bed utilization during the last 6 and 12 months of life, with a mean hospital stay of 32 and 40 days respectively.^39^ This was associated with high medical expenses and cost more than $120,000 per patient.^39^ Earle et al. and colleagues defined criteria for aggressive end-of-life care, which involved the number of admissions to emergency rooms, intensive care units and inpatient hospital wards during the last month, as well as the use of intravenous chemotherapy during the last 2 weeks of life.^81,82^ Based on these criteria, adolescents and young adults tend to have a high incidence of receiving aggressive care at the end of life, resulting in poor quality of end-of-life care.^37,39^

### Advance care planning

Studies in our scoping review that examined the opinions and preferences toward advance care planning showed that the majority of adolescents and young adults encouraged these discussions, and preferred their early introduction.^25,31^ There are different age-specific documents, created to help adolescents and young adults plan for their future medical needs, such as “Five Wishes,” “My Thoughts, My Wishes, My Voice,” and “Voicing My Wishes.”^30,31^ Further adaptation of these documents to match adolescents and young adults’ needs, together with introduction of advance care planning discussions early in the diagnosis and during treatment planning, can help in better understanding these patients’ needs, and improving the quality of their medical care.

### Strengths and limitations

To our knowledge, this is the first scoping review to report on the palliative care needs of adolescents and young adults with advanced illnesses, as a distinct age group. We conducted an extensive search of multiple bibliographic databases from inception to October 21, 2021. We also searched through the grey literature and identified studies through hand search of the references. Our inclusion criteria were inclusive to all study designs and types of advanced illnesses, which helped to capture the broad spectrum of evidence within the available literature.

However, our review has limitations. Due to the inconsistent definition of the adolescents and young adults age range across different healthcare systems, we only focused on the studies that specifically addressed adolescents and/or young adults. Further, our search excluded non-English language studies, and would not have included some of the studies that discussed palliative care components, but were not indexed as “palliative care.” Some of the findings in this review were obtained from case reports, and need further evidence to be generalized among the adolescent and young adult population.

## Conclusion

This scoping review provides an overview of the available literature related to the unique palliative and end-of-life care needs of adolescents and young adults with advanced illness. The reviewed literature paints a clear picture of disparity for this vulnerable and underserviced population as it relates to their high distress levels, unmet needs and poor quality of end of life care. Our findings show that adolescents and young adults are more likely to experience severe refractory symptoms, and receive intensive medical measures at end of life. Despite the concerns raised by these patients’ families, they are interested in engaging in age-appropriate advance care planning discussions. As such, there is a need for designated palliative care teams, with special age-focused training, to address this population’s complex needs. More prospective research centered around adolescents and young adults is needed, to ensure the provision of high-quality medical care to this age group.

## Supplemental Material

sj-pdf-1-pmj-10.1177_02692163221136160 – Supplemental material for Palliative care for adolescents and young adults with advanced illness: A scoping review Click here for additional data file. Supplemental material, sj-pdf-1-pmj-10.1177_02692163221136160 for Palliative care for adolescents and young adults with advanced illness: A scoping review by Mohamed Abdelaal, Jonathan Avery, Ronald Chow, Nasreen Saleem, Rouhi Fazelzad, Pamela Mosher, Breffni Hannon, Camilla Zimmermann and Ahmed al-Awamer in Palliative Medicine
