# Supplementary material for: Palliative care for adolescents and young adults with advanced illness: A scoping review
Source: Palliat Med. 2022 Nov 9;37(1):88–107. doi: 10.1177/02692163221136160 (PMC9841827; doi:10.1177/02692163221136160)
Supplement: sj-pdf-1-pmj-10.1177_02692163221136160 – Supplemental material for Palliative care for adolescents and young adults with advanced illness: A scoping review [file sj-pdf-1-pmj-10.1177_02692163221136160.pdf]

Ovid MEDLINE(R) ALL 1946 to October 21, 2021

#	Searches	Results	Type
1	Palliative Medicine/	440	Advanced
2	Palliative Care/	58233	Advanced
3	Hospice Care/	7116	Advanced
4	"Hospice and Palliative Care Nursing"/	1463	Advanced
5	Terminal Care/	29999	Advanced
6	Terminally Ill/	6690	Advanced
7	exp Advance Care Planning/	10285	Advanced
8	palliat*.tw,kf,kw.	84867	Advanced
9	hospice*.tw,kf,kw.	13927	Advanced
10	(advance* adj3 care*).tw,kf,kw.	15500	Advanced
11	(advance* adj3 plan*).tw,kf,kw.	7656	Advanced
12	(advance* adj3 disease?).tw,kf,kw.	50034	Advanced
13	(advance* adj3 ill*).tw,kf,kw.	1994	Advanced
14	(advance* adj3 stage?).tw,kf,kw.	59562	Advanced
15	(advance* adj3 phase?).tw,kf,kw.	4642	Advanced
16	(advance* adj3 sick*).tw,kf,kw.	81	Advanced
17	(terminal* adj3 patient?).tw,kf,kw.	8833	Advanced
18	(terminal* adj3 inpatient?).tw,kf,kw.	61	Advanced
19	(terminal* adj3 outpatient?).tw,kf,kw.	12	Advanced
20	(terminal* adj3 care?).tw,kf,kw.	3809	Advanced
21	(terminal* adj3 ill*).tw,kf,kw.	7910	Advanced
22	(terminal* adj3 sick*).tw,kf,kw.	45	Advanced
23	(terminal* adj3 disease?).tw,kf,kw.	2194	Advanced
24	(terminal* adj3 stage?).tw,kf,kw.	4023	Advanced
25	(terminal* adj3 failure?).tw,kf,kw.	1881	Advanced
26	(terminal* adj phase?).tw,kf,kw.	2251	Advanced

27	(terminal* adj mode?).tw,kf,kw.	47	Advanced
28	(end adj3 life).tw,kf,kw.	28764	Advanced
29	end-stage?.tw,kf,kw.	76584	Advanced
30	palliat*.jw.	25412	Advanced
31	or/1-30	354228	Advanced
32	Pain/	139238	Advanced
33	Pain Management/	37773	Advanced
34	Pain Measurement/	91047	Advanced
35	Symptom Assessment/	6490	Advanced
36	Psychosocial Support Systems/	808	Advanced
37	Psychometrics/	81431	Advanced
38	(pain? adj3 manag*).tw,kf,kw.	42083	Advanced
39	(pain? adj3 measur*).tw,kf,kw.	19892	Advanced
40	(pain? adj3 assess*).tw,kf,kw.	27362	Advanced
41	(pain? adj3 evaluat*).tw,kf,kw.	14688	Advanced
42	(pain? adj3 scale?).tw,kf,kw.	26076	Advanced
43	(pain? adj3 questionnaire?).tw,kf,kw.	8244	Advanced
44	(pain? adj3 prevalen*).tw,kf,kw.	5536	Advanced
45	(ache? adj3 manag*).tw,kf,kw.	18	Advanced
46	(ache? adj3 measur*).tw,kf,kw.	537	Advanced
47	(ache? adj3 assess*).tw,kf,kw.	113	Advanced
48	(ache? adj3 evaluat*).tw,kf,kw.	288	Advanced
49	(ache? adj3 scale?).tw,kf,kw.	7	Advanced
50	(ache? adj3 questionnaire?).tw,kf,kw.	3	Advanced
51	(ache? adj3 prevalan*).tw,kf,kw.	0	Advanced
52	((discomfort* or dis-comfort*) adj3 manag*).tw,kf,kw.	188	Advanced
53	((discomfort* or dis-comfort*) adj3 measur*).tw,kf,kw.	588	Advanced
54	((discomfort* or dis-comfort*) adj3 assess*).tw,kf,kw.	761	Advanced

55	((discomfort* or dis-comfort*) adj3 evaluat*).tw,kf,kw.	529	Advanced
56	((discomfort* or dis-comfort*) adj3 scale?).tw,kf,kw.	535	Advanced
57	((discomfort* or dis-comfort*) adj3 questionnaire?).tw,kf,kw.	227	Advanced
58	((discomfort* or dis-comfort*) adj3 prevalen*).tw,kf,kw.	123	Advanced
59	(symptom? adj3 manag*).tw,kf,kw.	15535	Advanced
60	(symptom? adj3 measur*).tw,kf,kw.	18538	Advanced
61	(symptom? adj3 assess*).tw,kf,kw.	29711	Advanced
62	(symptom? adj3 evaluat*).tw,kf,kw.	14072	Advanced
63	(symptom? adj3 questionnaire?).tw,kf,kw.	10920	Advanced
64	(symptom? adj3 prevalen*).tw,kf,kw.	12564	Advanced
65	(symptom? adj3 psych*).tw,kf,kw.	47182	Advanced
66	psychosocial*.tw,kf,kw.	107244	Advanced
67	psycho-social*.tw,kf,kw.	4571	Advanced
68	psychometric?.tw,kf,kw.	51930	Advanced
69	psycho-metric?.tw,kf,kw.	16	Advanced
70	or/32-69	604589	Advanced
71	31 and 70	22909	Advanced
72	exp animals/ not (exp animals/ and exp humans/)	4899511	Advanced
73	71 not 72	22761	Advanced
74	limit 73 to ("all infant (birth to 23 months)" or "newborn infant (birth to 1 month)" or "infant (1 to 23 months)" or "preschool child (2 to 5 years)" or "child (6 to 12 years)" or "middle age (45 to 64 years)" or "middle aged (45 plus years)" or "all aged (65 and over)" or "aged (80 and over)")	10567	Advanced
75	limit 74 to ("adolescent (13 to 18 years)" or "young adult (19 to 24 years)" or "adult (19 to 44 years)" or "young adult and adult (19-24 and 19-44)")	5603	Advanced
76	74 not 75	4964	Advanced
77	73 not 76	17797	Advanced

78	(young or young adult? or adolescen* or teenage* or teen-age* or TYA or AYA).tw,kf,kw.	807345	Advanced
79	73 and 78	617	Advanced
80	77 or 79	17855	Advanced
81	limit 80 to (clinical conference or consensus development conference or consensus development conference, nih or news or newspaper article or retracted publication or "retraction of publication")	173	Advanced
82	80 not 81	17682	Advanced
83	limit 82 to english language	16302	Advanced
